# Generic and Hearing-Specific Quality of Life in Older Adult Cochlear Implant Users

**DOI:** 10.7759/cureus.66042

**Published:** 2024-08-02

**Authors:** Norie Imagawa, Masaomi Motegi, Yuiko Kondo, Takashi Shimazaki, Takashi Yamauchi, Machi Suka

**Affiliations:** 1 Department of Health and Welfare, Prefectural University of Hiroshima, Hiroshima, JPN; 2 Department of Otolaryngology-Head and Neck Surgery, Gunma University Graduate School of Medicine, Gunma, JPN; 3 Department of Otorhinolaryngology, Jikei University Hospital School of Medicine, Tokyo, JPN; 4 Department of Public Health and Environmental Medicine, Jikei University School of Medicine, Tokyo, JPN

**Keywords:** profound hearing loss, hearing-specific quality of life, generic quality of life, older adults, cochlear implantation

## Abstract

Background

This study aimed to evaluate the quality of life (QOL) of older adults using cochlear implants (CIs) by focusing on how individual characteristics and speech perception are related to generic QOL ratings and utilizing the Health Utilities Index Mark III (HUI3) for assessment.

Methodology

A cross-sectional study was conducted with 19 participants aged ≥60 years, who were within one to five years post-implant activation. Data were obtained through self-administered questionnaires, including the HUI3 for generic QOL assessment and disease-specific indexes. Speech perception tests and chart reviews provided personal characteristic data. Statistical analysis was performed using the Mann-Whitney U-test, one-way analysis of variance, and Pearson’s correlation coefficient.

Results

In total, 18 patients responded to the study. This study revealed that the generic QOL in older adult CI users was lower than that in the general older adult population. There was no significant association between QOL and variables such as sex, duration of implant usage, or age. However, a longer duration of hearing loss before receiving an implant was associated with higher generic QOL scores. Additionally, a strong correlation was observed between the hearing-related QOL score and utility scores.

Conclusions

Longer pre-implantation hearing loss correlated with better post-implantation QOL. Additionally, a reduced QOL, specifically within the hearing attribute, a subdomain of the HUI3, was associated with a lower overall generic QOL. The results suggest that generic QOL cannot be evaluated based on hearing ability alone and that cochlear implantation does not completely improve generic QOL. This study represents an important first step in understanding the QOL of older adult CI users from a variety of backgrounds.

## Introduction

The global demographic is increasingly skewed toward an older population. Notably, Japan exhibits one of the highest aging rates globally, and this trend is projected to escalate. Hearing loss is a common health issue associated with aging. This condition, which often leads to communication challenges, significantly increases the risk of depression [1,2], dementia, and increased mortality [3]. Consequently, hearing loss in older adults is a critical determinant of the deterioration of their quality of life (QOL).

Concurrently, there is a growing interest in the potential of hearing aids to mitigate adverse health outcomes associated with age-related hearing loss, and recent cohort studies [4] have yielded insightful findings. Specifically, the use of hearing aids has been shown to reduce cognitive decline by 48% among the older population aged 70-84 years, a group particularly susceptible to such a decline. This suggests that effective hearing augmentation could not only arrest cognitive deterioration but also potentially forestall the early reduction in QOL associated with aging.

Cochlear implants are vital auditory support devices designed for individuals with profound hearing loss, who find little to no benefit from conventional hearing aids. As the global population ages, the number of older adults using cochlear implants is increasing, underscoring their effectiveness as a means of auditory supplementation. Despite requiring surgery and extensive rehabilitation, cochlear implants have demonstrably augmented speech perception, thereby significantly enhancing the QOL in older patients. Notably, the positive impact of cochlear implantation on the QOL of older individuals is well-documented [5-7].

QOL can be measured using various approaches, including disease-specific and general metrics. Cochlear implant users have been the focus of numerous QOL assessments globally, primarily utilizing disease-specific scales tailored to auditory impairments. Such scales, including the Glasgow Benefit Inventory (GBI) [8] and the Hearing Handicap Inventory for Adults (HHIA) [9], provide valuable insights into the specific challenges and changes experienced in conditions such as hearing loss. Hearing Handicap Inventory for the Elderly (HHIE) [10] is often used for older adults. However, a limitation of these disease-specific scales is their inability to facilitate comparisons between different diseases.

In contrast, broader general measures such as the Medical Outcome Study Short-Form (MOS SF-36) [11] and General Health Questionnaire (GHQ) [12] allow for such cross-disease comparisons. These assessments cover multiple dimensions of QOL, including physical, psychological, active, and social aspects. However, Arnoldner et al. reported that the SF-36 is not ideally suited for evaluating QOL in cochlear implant users [13,14].

Among general measures, those that allow utility values to be calculated from health status are called preference-based measures. Preference-based measures such as the EuroQOL (EQ-5D) [15], SF-6D [16], and Health Utilities Index Mark III (HUI3) [17,18] have been adopted in this context. Notably, the HUI3 incorporates crucial *hearing* and *cognition* components. Shiroiwa et al. indicated general correlations between the utility values of the EQ-5D and HUI3, with notable differences in patients with dementia, depression, hearing loss, and visual impairment [19]. The HUI3 allows the calculation of a quality-adjusted life-year index that accounts for both QOL improvements and extended survival years [20]. Therefore, the UK National Institute for Health and Care Excellence endorses the HUI3 as the most appropriate instrument for measuring QOL in people with hearing loss [21]. Consequently, HUI3 is considered suitable for assessing the generic QOL in the hearing-impaired population.

Surveys targeting older adult cochlear implant users should also include preference-based measures [6]. We advocate for the use of HUI3 as a more suitable preference-based assessment of QOL, particularly for older adults and those with hearing impairments. Historically, research in this field has focused predominantly on disease-specific metrics that address hearing impairment, thereby neglecting the holistic evaluation of QOL. Given that the HUI3 encompasses essential *hearing* and *cognitive* components, it is recognized as the most effective tool for assessing health-related well-being in cochlear implant users [13]. Nevertheless, the degree to which post-implantation hearing ability and personal attributes influence generic QOL in this demographic still requires thorough investigation [7], and the exploration of generic QOL values in older adult cochlear implant users is expected to yield valuable data. These data will be crucial for comparative analyses with the general older adult population and for the assessment of other diseases.

This study aimed to evaluate the generic QOL of older adults using cochlear implants utilizing the HUI3 values. We explored the relationship between individual characteristics and these utility values. Additionally, this study investigated the correlation between QOL within various HUI3 subdomains and overall QOL ratings to identify factors that significantly impact these utility values.

## Materials and methods

Study design and setting

This cross-sectional investigation using self-administered questionnaires for data collection was conducted at tertiary care hospitals affiliated with our university between March and August 2022. All eligible patients within the specified period were invited to participate. Participation was contingent upon providing written consent, and questionnaires were disseminated to consenting participants. The responses were collected either in person or by mail. Additionally, a retrospective review of the participants’ charts was conducted concurrently to collect personal characteristic data. This study was conducted in strict accordance with the Strengthening the Reporting of Observational Studies in Epidemiology (STROBE) guidelines. The study protocol was approved by the relevant institutional review board (approval number: 33-425(11050)). The initiation of the study was predicated on obtaining written informed consent from all participants, in compliance with the ethical standards outlined in the Declaration of Helsinki.

Study participants

This study included 19 patients aged 60 years or older who received cochlear implants between 2016 and 2021 and were attending tertiary care hospitals affiliated with our university. Among these patients, data from those aged 60 years or older who gave consent for the study were included. Building on observational studies [22] that reported the stability of hearing-specific health-related QOL from one to five years post-implant activation, this study focused exclusively on cases within this timeframe where QOL fluctuations were not anticipated. Eligibility for cochlear implantation was determined in accordance with the Japanese criteria, which necessitated severe bilateral hearing loss of 90 dB or more or moderate-to-severe loss of 70-90 dB, with speech intelligibility not exceeding 50% despite optimal hearing aid use. Patients with compromised decision-making capabilities, reading difficulties, pre-lingual deafness, and those who did not provide consent to participate in the study were excluded.

Participant Characteristics

The participants’ personal characteristics were retrospectively obtained through chart reviews. The clinical data of the participants, including age, sex, duration of cochlear implant usage, period of preoperative hearing loss, residential status, and employment status, were meticulously extracted. The period of preoperative hearing loss was the number of years since the person became aware of his/her hearing loss. Age groups were categorized into three distinct brackets, i.e., the 60s (encompassing ages 60-69 years), 70s (ages 70-79 years), and 80s (ages 80-89 years). The duration of preoperative hearing loss was defined as the interval from the onset of the patient’s self-awareness of hearing impairment to the point of receiving a cochlear implant.

Study Participants and Characteristics

During the study period, 20 patients, all aged 60 years or older, were initially selected for inclusion following their cochlear implantation. One individual was excluded because of a reading disability. Consequently, 19 patients were invited to participate in this study. Of these, 18 (94.7%) provided informed consent and actively responded.

Among the 18 participants in the study, nine were male and nine were female. The average age was 72.7 (standard deviation (SD) = 6.96) years. Regarding the age group, five were in their 60s, 11 were in their 70s, and two were in their 80s.

Measures

Hearing Outcome

Our investigation focused on speech perception tests conducted at six months post-implantation. The test battery employed was CI2004, which was specifically designed for cochlear implant users in Japan. CI2004 comprises evaluations of consonants, monosyllables, words, and sentences. The measurements were conducted in a soundproof environment with stimuli presented at a sound pressure level of 65 dB.

Disease-Specific Quality of Life

In this study, we utilized three distinct disease-specific indices, i.e., HHIE, Tinnitus Handicap Index (THI), and Dizziness Handicap Index (DHI). The participants were asked to reflect on their typical health state while completing the questionnaire.

HHIE, a hearing handicap-specific assessment comprising 25 items [10], was designed to evaluate subjective hearing impairment in older individuals. HHIE is divided into the following two subscales: social and emotional. The scores range from 0-16 (indicating no handicap), 18-42 (mild to moderate handicap), and 44 or more (severe handicap).

THI [23] is another 25-item assessment, measuring the impact of tinnitus on daily life. It employs a five-point scale, where 0-16 points suggest no handicap, 18-36 points mild handicap, 38-56 points moderate handicap, 58-76 points severe handicap, and 78-100 points indicate a catastrophic handicap.

Similarly, DHI [24] is a 25-item handicap-specific tool rated on a four-point scale. Scores are interpreted as follows: 0-14 points (no handicap), 16-25 points (mild handicap), 28-44 points (moderate handicap), and 46 points or more (severe handicap).

Preference-Based Quality of Life

In our study, we used the Japanese HUI3 [25] to determine the utility score for assessing the QOL in cochlear implant users. This questionnaire-based system, developed by the HUI Institute [17,18], requires participants to evaluate their current health status and typical health conditions. Notably, we adapted the questionnaire by substituting “hearing aid” with “cochlear implant.”

This system comprises the following three components: a questionnaire, classification, and scoring functions, which are collectively employed to calculate the utility score. Initially, the utility functions for eight attributes, i.e., vision, hearing, speech, ambulation, dexterity, emotion, cognition, and pain, were determined. Subsequently, the responses for each attribute are categorized based on the HUI framework. The utility values were derived from the level of each attribute using the Japanese scoring function. Notably, the “scoring function” adheres to the Japanese value sets [25,26]. Finally, the overall “utility score” was computed using the Japanese multi-attribute utility theory. This score generally ranges from 0-1, where 0 represents death, and 1 denotes perfect health. A score closer to 1 indicated a better health status.

Statistical analysis

Statistical analyses were performed to ascertain whether participant characteristics influenced utility scores. The Mann-Whitney U-test was used to compare scores between male and female participants. Additionally, the impact of age and duration of cochlear implant use on the utility score was assessed using the one-way analysis of variance.

The relationship between the utility score and duration of hearing loss, disease-specific handicap indices, and outcomes following cochlear implantation were evaluated using Pearson’s product-moment coefficient. Similarly, the correlation between the utility score and each domain’s utility function within HUI3 was analyzed using the same method. The correlation coefficient of 0 to less than 0.2 was defined as almost irrelevant, 0.2 to less than 0.4 as weakly correlated, 0.4 to less than 0.7 as moderately correlated, and 0.7 to 1 as strongly correlated.

All statistical analyses were performed using SPSS version 27.0.0 (IBM Corp., Armonk, NY, USA). Statistical significance was set at p-values < 0.05.

## Results

Study participants and characteristics

Table 1 and Table 2 present the demographic characteristics of the participants. The average age was 72.7 (SD = 6.96) years. At the time of their response, participants were within one to five years post-cochlear implantation, with an average (SD) duration of 3.2 (1.2) years. All participants consistently used a unilateral cochlear implant and received rehabilitation services from a speech-language-hearing therapist.

**Table 1:** Personal characteristics and condition scores of the study participants. *: CI2004 (Japanese open-set speech perception test battery) at six months post-implantation. Unity scores were calculated using the Health Utilities Index Mark III (HUI3); a unity score of 0 is indicated as dead and 1 as perfect health. HHIE = Hearing Handicap Inventory for the Elderly; THI = Tinnitus Handicap Index; DHI = Dizziness Handicap Index; QOL = quality of life

Characteristics and condition scores	Median	Interquartile range
Personal characteristics		
Age (years)	73.0	8.0
Duration of cochlear Implant use (years)	3.0	1.8
Duration of preoperative hearing loss (years)	19.0	11.3
Speech perception* (%)		
Monosyllable	40.0	35.5
Words	72.0	48.0
Sentences	77.0	49.0
Disease-specific QOL index		
HHIE score	52.0	17.0
THI score	14.0	26.0
DHI score	0.0	6.0
Health Utilities Index Mark III		
Vision	0.97	0.0
Hearing	0.74	0.18
Speech	1.00	0.07
Ambulation	1.00	0.0
Dexterity	1.00	0.0
Emotion	0.97	0.06
Cognition	1.00	0.09
Pain	0.96	0.04
Utility	0.63	0.17

**Table 2:** Participants’ demographics. *: CI2004 (Japanese open-set speech perception test battery) at six months post-implantation; **: Duration of cochlear implant (years); ***: Duration of preoperative hearing loss (years), ****: with, living with others. Unity scores were calculated using the Health Utilities Index Mark III (HUI3); a unity score of 0 is indicated as dead and 1 as perfect health. HHIE = Hearing Handicap Inventory for the Elderly; THI = Tinnitus Handicap Index; DHI = Dizziness Handicap Index; QOL = quality of life; N/A = not available

Number	Personal characteristics						Speech perception* (%)			Disease-specific QOL Index			Health Utilities Index Mark III								
	Age (y)	Sex	Implant use (years) **	Hearing loss (years) ***	Living status****	Employment	Monosyllable	Words	Sentences	HHIE score	THI score	DHI score	Vision	Hearing	Speech	Ambulation	Dexterity	Emotion	Cognition	Pain	Utility
1	62	M	2	28	With	No	13	13	16	82	0	6	0.97	0.70	1.00	1.00	1.00	0.94	1.00	1.00	0.64
2	63	M	4	23	With	No	70	88	100	54	N/A	N/A	0.97	0.70	1.00	1.00	1.00	0.77	0.90	0.88	0.41
3	65	F	4	20	With	No	18	72	83	36	4	0	0.97	0.70	0.93	1.00	1.00	1.00	1.00	1.00	0.63
4	65	F	3	24	With	No	50	84	97	70	0	0	0.97	0.70	1.00	1.00	1.00	0.94	1.00	1.00	0.64
5	68	F	3	18	With	No	25	40	43	64	28	4	0.97	0.88	0.93	1.00	1.00	0.94	1.00	0.96	0.72
6	70	M	5	11	With	Yes	0	0	0	48	6	0	0.97	0.70	0.76	1.00	1.00	1.00	0.90	1.00	0.46
7	71	M	5	8	With	No	18	8	7	54	14	48	0.97	0.78	1.00	0.94	1.00	0.77	1.00	1.00	0.55
8	71	M	3	15	With	No	30	44	40	74	16	0	0.97	0.70	0.76	0.94	1.00	1.00	0.74	0.88	0.31
9	72	M	2	3	With	No	40	77	86	54	26	30	0.97	0.88	1.00	1.00	1.00	0.94	0.93	0.96	0.72
10	74	F	3	14	With	No	75	88	92	54	26	18	0.97	0.70	1.00	1.00	1.00	1.00	0.93	0.96	0.61
11	74	M	3	19	With	Yes	N/A	N/A	N/A	48	26	0	0.97	0.88	1.00	1.00	1.00	1.00	0.90	1.00	0.77
12	74	F	2	52	Alone	No	32	28	32	40	54	4	1.00	0.70	1.00	1.00	1.00	1.00	1.00	0.96	0.67
13	75	F	4	26	With	No	78	88	87	52	0	2	0.97	0.88	0.76	1.00	1.00	0.94	1.00	1.00	0.61
14	77	F	5	18	With	No	N/A	N/A	N/A	44	36	96	0.97	0.88	0.76	1.00	1.00	1.00	0.90	0.96	0.56
15	77	M	3	29	With	No	87	92	87	26	0	0	1.00	0.88	1.00	1.00	1.00	0.94	1.00	1.00	0.83
16	78	F	4	19	With	No	30	68	53	32	6	0	0.97	0.88	0.93	1.00	1.00	1.00	1.00	0.96	0.76
17	85	M	1	5	Alone	No	55	76	77	30	20	0	1.00	0.54	1.00	1.00	0.94	1.00	1.00	0.96	0.49
18	88	F	1	39	With	No	43	72	47	18	0	0	0.97	0.88	1.00	1.00	1.00	0.94	1.00	0.96	0.77

The sex distribution among the participants was evenly split, with a 50-50 male-to-female ratio. Most participants were in their 70s, with no participants in their 90s or older. A significant majority (88.9%) of participants resided with family members, whereas 11.1% were employed. The median (interquartile range (IQR)) was established at 19.0 (11.3) years, indicating that 83.3% of the participants had a history of hearing loss for ≥10 years. Notably, for Cases 11 and 14, the assessment of speech perception using the CI2004 criteria was not feasible.

Disease-specific quality of life

The median HHIE score was determined to be 52.0 with an IQR of 27.0. While the median score falls within the severe handicap range according to the handicap index, individual HHIE scores ranged from 18 to 74, indicating that some subjects showed mild-to-moderate handicap. In contrast, the median THI score was 14.0 (26.0), and the median DHI score was 0.0 (6.0). Both of these latter scores fall under the no handicap classification of their respective handicap indices.

Preference-based quality of life in Health Utilities Index Mark III

Table 3 delineates the comparative analysis of generic social preference-based QOL scores derived from the HUI3 across various participant demographics. Our findings indicated no statistically significant correlation between the generic utility scores and variables such as sex (p = 0.297), duration of cochlear implant use (p = 0.739), or age (p = 0.977). However, owing to the skewed sample size, the analyses of cohabitation and employment status were deemed inconclusive.

**Table 3:** Comparison of the utility score on the Health Utilities Index Mark III based on participant characteristics. Unity scores were calculated using the Health Utilities Index Mark III (HUI3); a unity score of 0 is indicated as dead and 1 as perfect health. *: Mann–Whitney U test, **: One-way analysis of variance. IQR = interquartile range

Variables	n	Utility score (median)	IQR	P-value
Sex				0.297*
Male	9	0.55	0.13	
Female	9	0.64	0.11	
Duration of implant use (years)				0.739**
1–2	2	0.63	-	
2–3	3	0.67	-	
3–4	6	0.68	0.25	
4–5	4	0.62	0.27	
5–6	3	0.55	-	
Age (years)				0.977**
60–69	5	0.64	0.16	
70–79	11	0.61	0.21	
80–89	2	0.63	-	

Regarding age-wise median generic utility scores, participants in their 60s scored 0.64, those in their 70s scored 0.61, and participants in their 80s scored 0.63. In contrast, Shiraiwa et al. [21] reported median scores of 0.907, 0.875, and 0.776 for Japanese community residents in their 60s, 70s, and 80s, respectively. The cochlear implant users in this study had lower utility values than the community-dwelling older adults.

Relationship between utility score and disease-specific handicap index, duration of hearing loss, and hearing outcome

**Figure 1:**
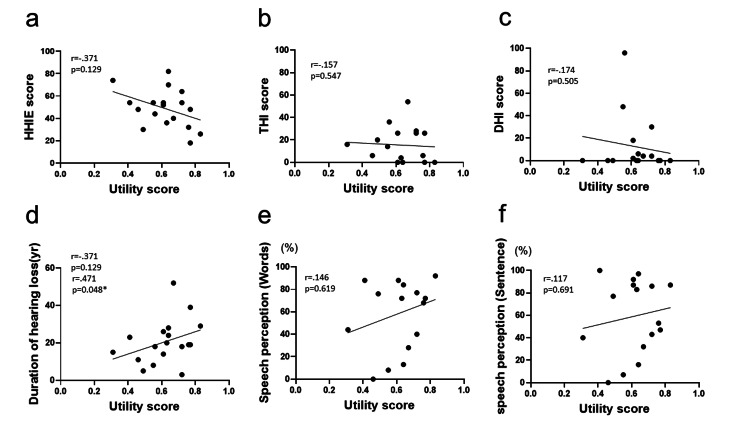
Relationship between generic quality of life (utility score) and disease-specific handicap indexes, duration of hearing loss, and hearing outcome. Unity scores were calculated using the Health Utilities Index Mark III (HUI3); a unity score of 0 is indicated as dead and 1 as perfect health. * p < 0.05. The duration of hearing loss (d) exhibits a significant positive correlation with the utility score. In contrast, HHIE (a), THI (b), DHI (c), and speech perception (words (e), sentences (f)) show no significant correlation with the utility score. HHIE = Hearing Handicap Inventory for the Elderly; THI = Tinnitus Handicap Index; DHI = Dizziness Handicap Index

Figure 1 presents a correlation plot analyzing the relationship between generic health-related QOL, as measured by utility scores and various factors, including hearing-specific handicap scores, duration of hearing loss, and auditory outcomes in words and sentences. The findings indicated that the utility scores did not show significant correlations with each of the following handicap scores: HHIE (r = -0.371, p = 0.129), THI (r = -0.157, p = 0.547), and DHI (r = -0.174, p = 0.505). Notably, in the case of the DHI score, while most cases fell within the range of 0-20, some approached scores near 100, leading to a substantial variance. A moderate yet significant correlation was observed between the utility score and duration of hearing loss (r = 0.471, p = 0.048), suggesting that a longer duration of hearing loss is associated with higher utility scores. However, no significant correlation was found between the utility scores and auditory outcomes for either word perception (r = 0.146, p = 0.619) or sentence perception (r = 0.117, p = 0.691). The range of hearing outcomes was notably broad among the individuals, with scores ranging from less than 10% to nearly 100%.

Relationship between utility score and utility function in eight attributes in Health Utilities Index Mark III

**Figure 2:**
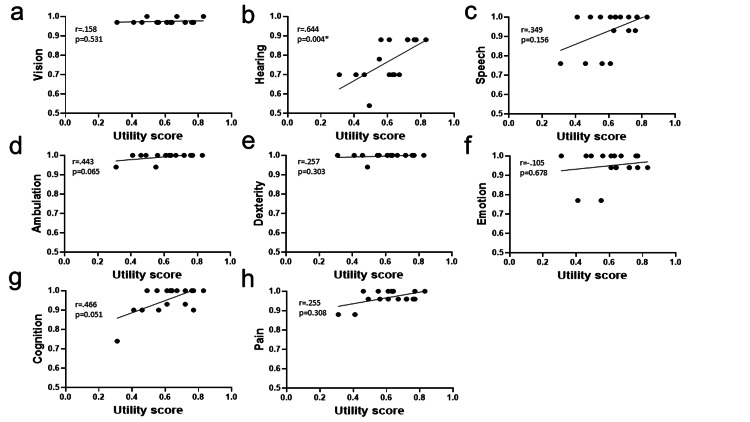
Relationship between generic quality of life (utility score) and utility function in each of the eight attributes in the Health Utilities Index Mark III. Unity scores were calculated using the Health Utilities Index Mark III (HUI3); a Unity score of 0 is indicated as dead and 1 as perfect health. *: p < 0.05. The utility function in the hearing attribute (b) shows significant moderate positive correlations with the utility score. Meanwhile, vision (a), speech (c), ambulation (d), dexterity (e), emotion (f), cognition (g), and pain (h) attributes exhibit no significant correlation with the utility score.

Figure 2 displays a correlation plot that examines the relationship between generic utility scores and each of the eight attributes in HUI3. In the context of this study’s cohort, a significant and strong correlation was exclusively observed between the hearing-related QOL score and utility scores (r = 0.644, p = 0.004). Notably, our analysis revealed the absence of significant correlations between generic utility scores and the other seven attributes within the HUI3 framework.

Individual profiles of poor quality of life cases

We present details of four cases where the utility score was below 0.5.

Case 2

Exhibiting the second-lowest generic utility score of 0.41, the patient’s speech perception was commendable (88% for words and 100% for sentences). However, a low hearing utility function score (0.70) and severe handicap score (54) on the HHIE were noted. Additionally, emotion and pain were rated at 0.77 and 0.88, respectively. The patient, who had bilateral cochlear otosclerosis requiring cochlear implant surgery, underwent frequent mapping adjustments to enhance speech perception. Despite good hearing ability, a significant difference was observed between the objective measures and subjective QOL. The patient expressed concerns regarding hearing impairment, limited job opportunities, and reduced enjoyment of music. Lower scores for emotion (0.77) and pain (0.88) were likely influenced by restrictions in economic and leisure activities and lumbar spinal stenosis, respectively.

Case 6

The patient had bilateral hearing loss for 11 years. A cochlear implant was placed in an ear that had lost hearing due to childhood mumps, accounting for approximately 60 years of hearing loss. While a hearing aid provided moderate improvement in the other ear, a cochlear implant in the ear with less auditory function permitted only basic sound detection and its limited hearing performance precluded effective measurements. This case was characterized by a severe HHIE (48) and a diminished hearing utility function (0.70), indicating significantly reduced QOL. Additionally, the chronic subdural hematoma caused speech difficulties, which further decreased the speech utility (0.76).

Case 8

An older adult cochlear implant user, the patient’s speech perception was below average (44% for words, and 40% for sentences). With a severe HHIE handicap score (74) and reduced hearing utility function (0.70), both subjective and objective speech perceptions were poor. The patient had a long-standing vestibular schwannoma in the non-implanted ear and experienced gradual hearing loss in the contralateral ear, which led to implantation. Additionally, the patient had a history of autoimmune antineutrophilic cytoplasmic antibody-associated vasculitis. Low scores for hearing, speech, cognition, and pain (0.76, 0.74, and 0.88, respectively) contributed to the lowest utility values in all cases.

Case 17

This patient, who received a cochlear implant in her 80s, started using hearing aids in her 70s due to chronic otitis media. Despite a mild-to-moderate HHIE score (30) and relatively good speech perception (76% for words and 77% for sentences), hearing utility function was the lowest at 0.54. Post-implantation, a tumor was discovered in the external auditory canal, necessitating multiple surgical procedures. Although speech perception did not deteriorate postoperatively, the patient reported dissatisfaction with the cochlear implant’s sound quality and difficulty in hearing everyday conversations. The HHIE revealed no difficulty with a score of 30 but indicated substantial issues with *face-to-face conversation* and *conversation with multiple people* in HUI3’s hearing item, resulting in a low utility score.

## Discussion

In this study, we assessed both generic and hearing-specific aspects of global QOL in older adult cochlear implant users using the HUI3. The HUI3 facilitated a comprehensive evaluation encompassing the entire spectrum of abilities and disabilities pertinent to a patient’s health status. Our findings revealed that the generic QOL of older adult cochlear implant users was inferior to that of their counterparts in the general older adult population within the groups. Additionally, we observed a noticeable trend: the poorer the QOL related solely to hearing impairment (as measured by HUI3), the lower the overall QOL. Conversely, the longer the duration of hearing loss pre-implantation, the better the overall QOL post-implantation.

Estimating patient satisfaction post-implantation remains challenging. The level of hearing can be perceived as an indirect indicator of hearing-specific QOL; however, the meta-analysis by McRackan et al. on cochlear implant users, particularly adolescents [27], reported only weak correlations between postoperative speech perception and disease-specific measures such as Speech, Spatial, and Quality, Nijmegen Cochlear Implant Questionnaire, and HHIE. In our current study utilizing HUI3, no correlation was observed between QOL scores and hearing outcomes post-implantation, reinforcing the notion that assessing speech perception alone is inadequate for determining QOL in older adult patients with CIs. Future research should explore whether the HUI3 provides a more precise and comprehensive evaluation of the QOL of older adults using cochlear implants than other hearing-specific QOL questionnaires.

To elucidate the social relevance of treatment, it is advantageous to use a preference-based scale for impact evaluation. This measure was employed in this study, allowing us to juxtapose cochlear implant users with their non-user peers in terms of QOL based on daily activities. Our data also indicate that cochlear implantation by itself does not fully compensate for the decline in general QOL resulting from hearing loss.

In contrast, self-assessments focusing solely on hearing attributes exhibited a moderate correlation with generic QOL scores in the HUI3 (r = 0.644). To evaluate the QOL of older adult cochlear implant users, it may be more effective to use the HUI3 questions, which potentially reflect their actual living conditions better than hearing-specific items in other questionnaires. This is partly because older adults are often unemployed, which makes questions about their everyday conversational situations more pertinent than those in a work setting. For instance, disease-specific scales such as the GBI, HHIA, and HHIE encompass queries about auditory and speech perception as well as the social and psychological impacts of challenging listening environments (e.g., meetings, telephone conversations, whispering). Conversely, HUI3 primarily inquires when respondents can hear “one-on-one conversations in silence” and “group conversations,” without setting up complex conversational scenarios such as phone calls or meetings. Hence, we believe that the HUI3 aligns more aptly with subjective handicap assessments, particularly in the listening environments of non-working older adult individuals. Our results suggest that for older adults, assessing subjective hearing QOL, which reflects everyday conversational handicaps, is crucial for evaluating the impact of cochlear implants.

A longer duration of hearing loss is associated with improved QOL. McRackan et al. employed Cochlear Implant-Related Quality of Life and QOL in a study involving cochlear implant users aged 18-89 years and found that prolonged hearing loss led to higher postoperative QOL scores [28]. This implies that adult cochlear implant users tend to exhibit increased post-implantation QOL scores correlated with the duration of their hearing loss, irrespective of the age at which they begin using the implants. This phenomenon may be linked to acceptance of disability. Individuals with hearing impairment undergo various stages from the onset of their condition to acceptance and seeking support [29,30]. Most participants in this study had post-lingual deafness, and many adopted hearing aids in their later years because of the progression of their hearing impairment from adolescence onwards, as hearing aids became less effective. It is conceivable that the heightened sense of well-being following the acquisition of auditory compensation through cochlear implants reflects prolonged acceptance of disability. Alternatively, the experience of navigating the disability acceptance process might contribute to a more favorable appraisal of QOL related to hearing, thereby enhancing the overall QOL, owing to the more effective utilization of cochlear implants for daily hearing challenges. The duration of hearing loss also impacted generic QOL.

The case-by-case validation of those with low utility scores on HUI3 revealed that low generic utility values do not necessarily correspond to significantly diminished speech perception or HHIE scores. Similar findings have been reported in other studies [21], indicating that a decrease in multi-attribute utility scores does not necessarily mirror deterioration in generic hearing. Various factors influencing health status, such as the cause and duration of hearing loss, level of demand for cochlear implants due to employment and hobbies, and comorbidities, were found to adversely affect the generic QOL.

Limitations

It is important to acknowledge certain limitations in the interpretation of our findings. First, we did not explore the relationship between pre-implantation hearing levels or QOL and post-implantation QOL data. In addition, the QOL survey was conducted in all cases one year after implantation; however, there were some cases where the evaluation of auditory performance was conducted less than one year post-implantation. Second, participants of the present study were restricted to a single center, possibly introducing selection bias regarding surgical indications. Third, study participants were limited to Japanese patients; thus, caution should be exercised when generalizing the present findings to non-Japanese populations. Finally, this study did not consider other potential relationships affecting hearing loss history and related factors.

## Conclusions

In this study, we assessed generic QOL in cochlear implant users aged 60 years and above, utilizing the HUI3. In contrast to younger people, older adults have individual differences in their physical and social backgrounds. Therefore, it is necessary to identify the factors that influence generic QOL. Notably, a longer duration of hearing loss before implantation was associated with improved generic QOL post-implantation. Additionally, a reduced QOL, specifically within the hearing attribute, a subdomain of the HUI3, was associated with a lower overall generic QOL. This study represents an important first step in understanding the QOL of older adult cochlear implant users from a variety of backgrounds.
